# Correction: A framework for testing independence between lane change and cooperative intelligent transportation system

**DOI:** 10.1371/journal.pone.0249804

**Published:** 2021-04-05

**Authors:** Mohammed Elhenawy, Sébastien Glaser, Andy Bond, Andry Rakotonirainy, Sébastien Demmel, Mahmoud Masoud

In the Abstract, there are errors in the second and fifth sentences of the paragraph. The correct sentences are:

The Queensland Department of Transport and Main Roads (TMR) are preparing for the Ipswich Connected Vehicle Pilot, the largest Field Operational Test (FOT) in Australia to evaluate C-ITS safety benefits. As part of supporting TMR deliver this Pilot,

In the planned FOT, the participating vehicles are only equipped with the vehicle C-ITS. However, we are investigating the usage of the Inertial Measurement Unit (IMU) as a second source of measurement (supporting device) based on the UAH-DriveSet dataset [26].

The following information is missing from the Acknowledgements: The Ipswich Connected Vehicle Pilot is an initiative of the Queensland Department of Transport and Main Roads, and is supported by the iMOVE CRC, the Motor Accident Insurance Commission, the Queensland University of Technology, Ipswich City Council, Telstra and the Federal Department of Infrastructure, Transport, Cities and Regional Development.
