# Supplementary material for: A framework for testing independence between lane change and cooperative intelligent transportation system
Source: PLoS One. 2020 Feb 27;15(2):e0229289. doi: 10.1371/journal.pone.0229289 (PMC7046226; doi:10.1371/journal.pone.0229289)
Supplement: S1 Dataset — (DOCX) [file pone.0229289.s001.docx]

**Data set dictionary**

- The first column is the label (0 is lane-change and 1 is lane-keeping)
- The second column is the trip id
- Columns from 3 to 33 the acceleration measurements in Y filtered by KF (Gs) within the 2w+1 window
- Columns from 34 to 64 are the acceleration measurements in Z filtered by KF (Gs) within the 2w+1 window
- Columns from 65 to 95 are the time series of the roll measurements (degrees) within the 2w+1 window
- Columns from 96 to 126 are the time series of the pitch measurements (degrees) within the 2w+1 window
