# Supplementary material for: A framework for testing independence between lane change and cooperative intelligent transportation system
Source: PLoS One. 2020 Feb 27;15(2):e0229289. doi: 10.1371/journal.pone.0229289 (PMC7046226; doi:10.1371/journal.pone.0229289)
Supplement: Attachment — Submitted filename: Rebuttal letter.docx [file pone.0229289.s003.docx]

**Comments to the Author**

1. Is the manuscript technically sound, and do the data support the conclusions?

The manuscript must describe a technically sound piece of scientific research with data that supports the conclusions. Experiments must have been conducted rigorously, with appropriate controls, replication, and sample sizes. The conclusions must be drawn appropriately based on the data presented.

Reviewer #1: Partly

Reviewer #2: Yes

Reviewer #3: Yes

2. Has the statistical analysis been performed appropriately and rigorously?

Reviewer #1: No

Reviewer #2: Yes

Reviewer #3: Yes

**Response :** Thank you.

3. Have the authors made all data underlying the findings in their manuscript fully available?

The [PLOS Data policy](http://www.plosone.org/static/policies.action#sharing) requires authors to make all data underlying the findings described in their manuscript fully available without restriction, with rare exception (please refer to the Data Availability Statement in the manuscript PDF file). The data should be provided as part of the manuscript or its supporting information, or deposited to a public repository. For example, in addition to summary statistics, the data points behind means, medians and variance measures should be available. If there are restrictions on publicly sharing data—e.g. participant privacy or use of data from a third party—those must be specified.

Reviewer #1: No

Reviewer #2: No

Reviewer #3: Yes

**Response :** Thank you. the pre-processed data in excel sheet is provided and attached with the documents. Also, the raw (the UAH-DriveSet ) data is available publically at: <http://www.robesafe.uah.es/personal/eduardo.romera/uah-driveset/#download>

The raw data is big size to be attached (3.3GB).

Please see Reference [26] in the manuscript

[26] E. Romera, L. M. Bergasa, and R. Arroyo, "Need data for driver behaviour analysis? Presenting the public UAH-DriveSet," in 2016 IEEE 19th International Conference on Intelligent Transportation Systems (ITSC), 2016, pp. 387-392.

4. Is the manuscript presented in an intelligible fashion and written in standard English?

PLOS ONE does not copyedit accepted manuscripts, so the language in submitted articles must be clear, correct, and unambiguous. Any typographical or grammatical errors should be corrected at revision, so please note any specific errors here.

Reviewer #1: No

Reviewer #2: Yes

Reviewer #3: Yes

**Response :** Thank you. The manuscript has been improved accordingly.

**5. Review Comments to the Author**

Please use the space provided to explain your answers to the questions above. You may also include additional comments for the author, including concerns about dual publication, research ethics, or publication ethics. (Please upload your review as an attachment if it exceeds 20,000 characters)

**Reviewer #1**:

**Comment 1.1:** This paper studies the safety benefits of C-ITS. More precisely, under slow/stopped vehicle (SSV) and road works warning (RWW) conditions, whether C-ITS warning displayed on the HMI helps the driver make lane changing decision? This is a quite practical question. Even though the answer is probably positive, we still need concrete evidence.

**Response 1.1:** Thank you. In order to clarify the merit of the paper we need to describe the problem we have in our FOT. The planed FOT has two safety use-cases(i.e. slow/stopped vehicle (SSV) and road works warning (RWW)) which display warning to the driver. We hypotheses that a remarkable percentage of the driver will change lane early upon seeing the HMI message. On the other hand the drivers who have the HMI off will change lanes early by chance. In other words, the percentage of early lane change in the C-ITS is higher than the percentage of early lane change in the Non-C-ITS.

The used vehicles are not equipped with a camera that can be used to detect the lane change so that we developed this framework. The main goal of the framework is testing the above hypotheses using the IMU measurement only. Please see Line 195-203.

**Comment 2.1:** The authors first train a random forest classifier to detect lane changes using publicly available dataset UAH-DriveSet, which contains raw IMU readings and corresponding lane change list. However, the authors do not mention where the C-ITS warning data come from. Their own experimental work or public dataset? Therefore, we could not validate the results on dependency of C-ITS warning message and lane change.

**Response 2.1:** Thank you. We should highlight the following

1. The data used in this paper is real data (the UAH-DriveSet).
2. The details of the traing and testing the classifiers is decribed in the paper, Lines 398-409 as follows

“ We used leave-one-out-cross-validation to divide the data into a training set and a testing set. Where in each fold we keep all the examples belonging to one trip as a test dataset and all other trips are used as the training dataset. The training datase is used to built the RF model which is tested using the unseen testing dataset. Then the classifcation outputs for the examples in the testing trip are saved. For each trip, we repeat this sequence of trainning-testing sequence and save the classification outputs. Finally, the classification outputs of all trips (i.e. the whole dataset) and the ground truth labels are used to calculate the True Positive Rate (TPR) and the False Positive Rate (FPR) where lane change is coded as positive.”

3. The classofcation output of all trips and the ground truth are used to simulate data collected from C-ITS as mentioned in the paper, Lines 425-434.

“The dataset has 3994 lane change examples and almost 4000 lane keeping examples. In addition to the ground truth labels of these examples, we have the hard decision and soft decision classification output from the RF. We set up the experiment as shown in Figure 7. We assume the sample size of the Non-C-ITS is $2M$ where randomly draw $M$ lane change examples and $M$ lane keeping examples without replacement. In the case of the C-ITS row, we randomly draw $M+k$ lane change examples and $M-k$ lane keeping examples without replacement. This makes the C-ITS examples equal the Non-C-ITS examples equal $2M$. Notice that, for the randomly chosen examples, if we summed the ground truth label it gives us the numbers shown in Figure 7.”

4. In the experimental work we checked the suitability of the framework for different sample size and effect size by changing the M and K parameters. Please see Statistical testing Section.
5. The data collection of our FOT will end late in 2020 and we will apply this framework to test the lane change hypotheses. Please see Line 150

**Comment 3.1:** The authors provide too many detailed statistic analyses, which distracts the reader's attention from the topic of the paper. The organization of the paper and the language usage should be further improved.

**Response 3.1:** Thank you. The statistics are importatnt for researchers and practitioners to understand the framework and consequently apply it to similar prblems.

Based on the reviewer comment, the paper has been revised accordingly.

**Reviewer #2**:

The authors proposed a framework to check the independency between lane changing and C-ITS. They train a random forest to precess data
Collected from IMU only and then developed a permutation test.

Some concerns:

**Comment 1.2:** Line 33, should it be Integrated surface … act instead of program?

**Response 1.2**: Thank you, we fixed the typo

**Comment 2.2:** Line 50, what compass4d stands for?

**Response 2.2**: Thank you. Compass4D stands for Cooperative Mobility Pilot on Safety and Sustainability Services for Deployment. We modified the manuscript accordingly.

**Comment 3.2:** Line 51, what sim-td stands for?

**Response 3.2:** Thank you. Safe and Intelligent Mobility - Test Field (SIM-TD) in Germany

**Comment 4.2:** Line 52-53, how to use lane change as a safety indicator.

**Response 4.2: Thank you.** As mentioned in the paper, Line 52-55, we are using lane change and early lane change interchangeably. Early lane change is a safety indicator where the HMI warning early informs the driver about a lane closure. Hence, the driver has enough time to change lane probably. More information can be found in recent research that uses the lane change as a safety indicator to evaluate the safety benefits of the Cooperative Intelligent Transportation Systems (C-ITS) [10].

[10]	Mohammed Elhenawy, Andy Bond and Andry Rakotonirainy, "C-ITS Safety Evaluation methodology based on Cooperative Awareness Messages," presented at the 21st IEEE International Conference on Intelligent Transportation Systems, Maui, Hawaii, USA, 2018.

**Comment 5.2:** Line 53, the authors have already mentioned c-its above. Here should be shorted form

**Response 5.2:** Thank you, we removed the redundancy

**Comment 6.2:** Line 54, the comma after learning should be deleted

**Response 6.2:** Thank you, we removed the comma

**Comment 7.2:** Line 57 and 61, whether use detection/prediction or prediction/detection, not both.

**Response 7.2:** Thank you, we changed the prediction/detection to detection/ prediction for the sake of consistency.

**Comment 8.2:** related work about lane change detection is a bit redundant

**Response 8.2:** Thank you. We did the related work section to show that this paper is different from the earlier research. To the best of knowledge, we are the first who proposed a framework to test the independence between the lane change and the C-ITS warning without collecting many data modalities to train accurate and sensitive machine learning classifier.

**Comment 9.2:** line 136 - line 141, the contribution could be clearer.

**Response 9.2:** Thank you. We made it clearer

**Comment 10.2:** Line 159-160, what do the authors mean with this sentence?

**Response 10.2:** Thank you. We replaced “The Slow / Stopped Vehicle (SSV) event provides warning to the Subject Vehicle (SV) (upstream) because of slow or stops a cooperative Target Vehicle (TV) (downstream)”

By

“The Slow / Stopped Vehicle (SSV) event provides warning to the Subject Vehicle (SV) (upstream) which is triggered by a slow or stopped cooperative Target Vehicle (TV) (downstream).”

**Comment 11.2:** Line 161, will “ CAM (cooperative awareness message)” be more consistent?

**Response 11.2:** Thank you. fixed

**Comment 12.2:** Line 170, consistency

**Response 12.2:** Thank you. Fixed

**Comment 13.2:** Line 179, can the author make the similarity and difference between stopped vehicle and lane closure clearer?

**Response 13.2:** Thank you for the constructive commnet. We added the following paragraph in the manscurpit, Line 184-189 to address the reviewer comment

“In the Non-C-ITS environment, the Stopped Vehicle is similar to lane closure due to road work because both of them cause safety risk and traffic capacity drop. In the case of lane closure due to road work, the driver will see the posted warning before the position of the lane closure but this kind of warning does not exist in the stopped car case. In the above two cases the event speed is zero however the speed of slow vehicle event is not zero. Thus, to our mind, it has less safety risk compared to the zero speed safety events. ”

**Comment 14.2:** Line 248, the author only use one sensor, IMU

**Response 14.2:** Thank you. More clarification has been added in Line 155-157as following: An inertial measurement unit (IMU) is an electronic device that uses a combination of [accelerometers](https://en.wikipedia.org/wiki/Accelerometer), [gyroscopes](https://en.wikipedia.org/wiki/Gyroscope), and sometimes [magnetometers](https://en.wikipedia.org/wiki/Magnetometers). Thus we get several measurements including the four channels mentioned in the paper;

1. longitudinal acceleration
2. lateral acceleration
3. Roll
4. Yaw

**Comment 15.2:** Line 254, what does “w” stand for?

**Response 15.2:** Thank you. More clarifications have been added to manuscript. We used w to describe the short window of length (2w+1) time stamps within which the lane change happened

For example assume w=5 then the length of this short window will be 11 as shown below

| 1 | 2 | 3 | 4 | 5 | 6 | 7 | 8 | 9 | 10 | 11 |
| --- | --- | --- | --- | --- | --- | --- | --- | --- | --- | --- |
|  |  |  |  | w | w+1 | w+2 |  |  |  | 2w+1 |

**Comment 16.2**: Line 257, will slide the window in different direction reverse the time series and impact the data?

**Response 16.2:** Sliding the window will not reverse the time series but will impact the data chosen as an example for the lane change. This data impact has the following two advantages;

1. The generated data set will have the event at different locations within the window and hence the model built using this data will be independent of the exact location of the event within the window.
2. We overcome the problem of relatively small number of events in the UAH-DriveSet.

For the illustration purpose assume that we have a time series its length is N and the lane change event is at time index 8. Moreover, the window length is 5. Shifting the window left and right will create several examples from the one event at time index 8 as shown in the figure below

Lane change event

Shift left

Shift right index

| 1 | 2 | 3 | 4 | 5 | 6 | 7 | 8 | 9 | 10 | 11 | 12 | 13 | 14 | $\cdots$ | N |
| --- | --- | --- | --- | --- | --- | --- | --- | --- | --- | --- | --- | --- | --- | --- | --- |
| 1 | 2 | 3 | 4 | 5 | 6 | 7 | 8 | 9 | 10 | 11 | 12 | 13 | 14 | $\cdots$ | N |
| 1 | 2 | 3 | 4 | 5 | 6 | 7 | 8 | 9 | 10 | 11 | 12 | 13 | 14 | $\cdots$ | N |
| 1 | 2 | 3 | 4 | 5 | 6 | 7 | 8 | 9 | 10 | 11 | 12 | 13 | 14 | $\cdots$ | N |
| 1 | 2 | 3 | 4 | 5 | 6 | 7 | 8 | 9 | 10 | 11 | 12 | 13 | 14 | $\cdots$ | N |

Time stamp

**Comment 17.2**: All the tables, could be polished to be reader-friendly

**Response 17.2:** Thank you, more clarfications have been done and we are open for any suggestion to further improve them.

**Comment 18.2**: All the figures could be clearer. A vague figure damages the originality in first impression

**Response 18.2:** Thank you. We improved the quality of the blurred figures.

**Comment 19.2**: line 357, used -> use
**Response 19.2:** Thank you. Fixed.

**Reviewer #3**:

The paper proposes and implements a framework for statistically testing the impact of C-ITS on lane change. Although such testing will be limited in scope in a fuller evaluation, the paper is informative and merits publication with minor revisions as follows:

**Comment 1.3**: line 65: Is there a reason to have C-ITS checking (in addition) for aggressive drivers coming from behind?

**Response 1.3:** Thank you.We meant to indicate that lane changing is complicated manouver because, safely changing lane in traffic requires the driver to look forward and to adjacent lanes.

**Comment 2.3**: figure 4, p. 11: Add "Total" as a header of the last column.

**Response 2.3:** Thank you. We added the header total.
**Comment 3.3**: line 349: How many times was the permutation run?

**Comment 3.3**: Thank you. 1000 times. We edited the text and stated the number of the permutations, please see Line 448.

**Comment 4.3**: line 395: Define M and k.

**Response 4.3:** Thank you. M and K have been defined in Line 425-434. In order to test the adopted framework for different sample size and effect size we defined the sample size and the effect size using M(number of lane change examples in Non- C-ITS) and K (deviation number from M) as shown in figure 7.

Where

2M is the total sample size of the C-ITS

2M is the total sample size of the Non-C-ITS

4M is the total sample size

the effect size is defined as $\pi_{Non-C-ITS}-\pi_{C-ITS}=\frac{M}{2M}-\frac{M+k}{2M}=\frac{-k}{2M}$

As shown in the above figure, the difference between the number of lane change and the number of lane keeping is 2K in the C-ITS case.

**Comment 5.3**: figure 7, p. 19: Add "Total" label for the marginal totals.

**Response 5.3:** Thank you we added the header total.
**Comment 6.3**: line 485: It would be useful to check the framework suitability with real data once collected.
**Response 6.3:** Thank you. We should highlight the following

1. The data used in this paper is real data
2. In the experimental work we checked the suitability of the framework for different sample size and effect size by changing the M and K parameters.
3. The data collection of our FOT will end late in 2020 and we will apply this framework to test the lane change hypotheses.

**Comment 7.3**: Please conduct a thorough language editing in a revised manuscript. This would help readability of the paper.

**Response 7.3:** Thank you. Based on the reviewer comment, we revised the language of the paper.
