# Supplementary material for: A framework for testing independence between lane change and cooperative intelligent transportation system
Source: PLoS One. 2020 Feb 27;15(2):e0229289. doi: 10.1371/journal.pone.0229289 (PMC7046226; doi:10.1371/journal.pone.0229289)
Supplement: Attachment — Submitted filename: Resonse Letter.docx [file pone.0229289.s004.docx]

**Response Letter for “PONE-D-19-28884”**

**Reviewer#1, Concern # 1**: Line 18: however?

**Author response:** Thank you, fixed.

**Reviewer#1, Concern # 2**: L 30: too many specific countries.

**Author response:** Thank you. We reduced the number of the specific countries that we mentioned as an example of countries involved in the research and development of ITS.

**Reviewer#1, Concern # 3**: L 37: what do CEN, ISO, and ETST stand for?
**Author response:** Thank you, fixed. We replaced “The interaction between ITS components is based on several standards such as CEN[2], ISO[3], ETST [4], depending on the jurisdiction.” By “The interaction between ITS components is based on several standards developed by different organizations such as European Committee for Standardization (CEN) [2], the International Organization for Standardization (ISO) [3], the European Telecommunications Standards Institute (ETST) [4], depending on the jurisdiction.”

**Reviewer#1, Concern # 4**: L 46: Is it necessary to list many projects?

**Author response:** Thank you. To our mind, listing these projects is important to show the widespread of these projects around the world. Moreover, the interested reader can find references to these important projects.

**Reviewer#1, Concern # 5**: L 72: what does LIDAR stand for?

**Author response:** Thank you. LIDAR stands for “LIght Detection and Ranging”. We updated the paper accordingly

**Reviewer#1, Concern # 6**: Please keep the reference style consistent through the whole script.

**Author response:** Mahmoud task PLEASE CHECK

**Reviewer#1, Concern # 7**: L 109; state of THE art.

**Author response:** Thank you, fixed

**Reviewer#1, Concern # 8**: L 112: proposed

**Author response:** Thank you, fixed

**Reviewer#1, Concern # 9**: L121: remove,.

**Author response:** thank you, fixed.

**Reviewer#1, Concern # 10**: L128: use the abbreviated version.

**Author response:** Thank you, fixed. We replaced “the three raw physiological signals” by “the ECG, GSR and RR signals.”

**Reviewer#1, Concern # 11**: So many existing works are listed with excess details

**Author response:** We mentioned much-existing work with some details to show that the previous work used data modalities that are not available in our FOT.

**Reviewer#1, Concern # 12**: The weakness of existing works

**Author response:** When we described much existing work, our goal was to clarify that they used different data modalities that are not available in our case. Moreover, to the best of our knowledge, we are the first who proposed using data collected from the C-ITS and the IMU to test the independence between lane change and cooperative intelligent transportation system without using other data modalities such as videos.

**Reviewer#1, Concern # 13**: Why the proposed framework is better for others.

**Author response:** As mentioned at the end of the introduction section “The present paper is different from earlier research in that it developed a framework consisting of the following:

1- Training a lane change/lane-keeping machine learning classifier using a limited number of sensing modalities (i.e. longitudinal acceleration, lateral acceleration, roll, and yaw)
2- Overcoming the uncertainty of machine learning classifier if it classifies one data point by summing up the output probabilities of the trained classifier of the whole collected data (i.e. all data points) to construct the 2x2 contingency table.
3- Estimating the null hypothesis of the test statistic using the proposed permutation test.

This framework enables us to test the independence between the lane change and the C-ITS warning without collecting expensive data modalities such as videos to train accurate machine learning classifier. In other words, the proposed framework allows us to test the hypotheses that assume C-ITS warnings at safety-critical situation help the drivers changing lane safely compared to the drivers who do not receive C-ITS warnings.”

**Reviewer#1, Concern # 14**: L138: a classifier for what? Should be specific.

**Author response:** Thank you, fixed. A classifier for lane change/lane-keeping.
**Reviewer#1, Concern # 15**: L 140: hard to understand.

**Author response:** We replaced the word pooling by the word summing up to make the statement more clear.

**Reviewer#1, Concern # 16**: L 143: statement is too general.

**Author response:** We made it more specific “This framework enables us to test the independence between the lane change and the C-ITS warning without collecting expensive data modalities such as videos to train accurate machine learning classifier. In other words, the proposed framework allows us to test the hypotheses that assume C-ITS warnings at safety-critical situation help the drivers changing lane safely compared to the drivers who do not receive C-ITS warnings.”

**Reviewer#1, Concern # 17**: Use abbreviated versions after explained. For example, L 164 and many other places.

**Author response:** Thank you, fixed.

**Reviewer#1, Concern # 18**: L224: Description of dataset is too long.

**Author response:** Thank you, fixed. We reduced the description of the dataset
**Reviewer#1, Concern # 19**: L 252: a noun is not a sentence.

**Author response:**

**Reviewer#1, Concern #20**: How could a random forest DOES NOT suffer from overfitting?

**Author response:** Thank you. In the random forest subsection, we stated that “The most significant advantage of the RF approach is that it does not suffer from overfitting because of the Law of Large Numbers, where the error rate of RF will get smaller as more CARTs are added. By adding more CATRs, the error rate will be saturated but will never get larger no matter how many CARTs are added.”

**Reviewer#1, Concern #21**: L 274: Have the authors proofread this script before submitting?

**Author response:** Yes, thank you.
**Reviewer#1, Concern #22**22. L 277: of and?

**Author response**: thank you, fixed
**Reviewer#1, Concern #23**: L 284: its length?

**Author response**: thank you,fixed

**Reviewer#1, Concern #24:** L 345: dds?

**Author response**: thank you, fixed
**Reviewer#1, Concern #25:** L 510: use words rather than variables in conclusion.
**Author response**: thank you, fixed.

**Reviewer #2:** The revised manuscript "A Framework for Testing Independence between Lane Change and Cooperative Intelligent Transportation System" has addressed my earlier comments. I have no other comments to submit.

**Author response**: thank you.

 6. PLOS authors have the option to publish the peer review history of their article ([what does this mean?](https://journals.plos.org/plosone/s/editorial-and-peer-review-process#loc-peer-review-history)). If published, this will include your full peer review and any attached files.


If you choose “no”, your identity will remain anonymous, but your review may still be made public.


**Do you want your identity to be public for this peer review?** For information about this choice, including consent withdrawal, please see our [Privacy Policy](https://www.plos.org/privacy-policy).

Reviewer #1: No

Reviewer #2: No
